# Efficacy of Topical Epinastine Eyelid Cream for Perennial Allergic Conjunctivitis: A Prospective Study

**DOI:** 10.7759/cureus.85364

**Published:** 2025-06-04

**Authors:** Hayato Tanaka, Mao Tanabe, Ayaka Kawaguchi, Hitoshi Tabuchi, Atsuki Fukushima

**Affiliations:** 1 Ophthalmology, Tsukazaki Hospital, Himeji, JPN

**Keywords:** 5-d itch scale, allergic conjunctivitis, antihistamine eyes drops, eye cream, proactive preventive care, qol questionnaire, transdermal drug delivery system

## Abstract

Introduction: Antihistamine eye drops are first-line treatments for allergic conjunctivitis; however, patient compliance and ocular itch exacerbation during medication intervals remain challenging. This study investigated the clinical application of 0.5% topical epinastine eyelid cream in patients with symptomatic perennial allergic conjunctivitis already using antihistamine eye drops.

Methodology: Fourteen patients with perennial allergic conjunctivitis using antihistamine eye drops were prescribed 0.5% topical epinastine eyelid cream. Patients applied the cream once daily before bedtime. Questionnaires assessed medication compliance, 24-hour ocular itch scores, and daytime quality of life (QOL). Statistical analysis used a mixed-effect model for repeated measures.

Results: All 14 patients completed the proactive eyelid cream medication. Ocular itch scores significantly decreased from baseline to the next morning (*P *= 0.003). Daytime QOL scores showed significant changes between baseline and first and second applications (*P *< 0.001 for both).

Conclusions: A 0.5% topical epinastine eyelid cream demonstrates potential as a convenient, once-daily treatment for allergic conjunctivitis, offering consistent symptom control and improved patients' QOL. Further long-term studies are recommended to validate these initial findings.

## Introduction

Antiallergic eye drops have been one of the first-line treatments for seasonal allergic conjunctivitis (SAC) and perennial allergic conjunctivitis (PAC) [1]. Patients with allergic conjunctivitis often use antiallergic eye drops to relieve ocular itching during symptomatic episodes [2]. On the other hand, Fukushima et al. reported that proactive and regular-time use of antihistamine eye drops before symptoms improves patients' quality of life (QOL) with SAC better than as-needed use [3]. The possible reason is the relevance of QOL and the frequency of ocular itches. As-needed use naturally experiences more frequent bouts of itching than proactive and regular-time use because patients with as-needed use apply eye drops after feeling itchy. Fukagawa et al. reported that QOL showed a stepwise decline corresponding to higher frequencies of itching [2].

The challenge of proactive use is that patients have to apply eye drops even when they do not have symptoms. In Japan, most antiallergic eye drops require two to four applications daily. Compliance rates for proactive antihistamine medication vary across previous studies. Fukushima et al. reported that the compliance rate of twice-daily eye drops was significantly higher than the rate of fourth-daily eye drops [4]. The report suggested that a lower frequency of applications can be the key to improving compliance with proactive medication. However, the rate of twice-daily eye drops was still low - 22.3% - and has room for improvement. The most common reason for not implementing proactive medication was that the patient would forget to take the drops (46%), followed by the difficulty of taking the drops during the day at work or school (20%).

Topical epinastine 0.5% eyelid cream is a novel treatment option for allergic conjunctivitis. It requires once-daily administration to the upper and lower eyelids. Epinastine is an H1-receptor antagonist and mast cell stabilizer, classified as an anti-allergic agent. The 0.5% topical epinastine eyelid cream delivers the drug slowly and percutaneously to the conjunctiva, helping to minimize fluctuations in antihistamine concentration within the conjunctival tissue [5]. The aim of 0.5% topical epinastine eyelid cream is to make proactive antihistamine medication easier for patients. Fujishima et al. performed a conjunctival allergen challenge (CAC; Ora, Inc., Andover, MA) test to compare 0.5% topical epinastine eyelid cream or placebo cream [6]. The CAC test observed that mean itch scores and mean conjunctival hyperemia scores were lower in the eyes 24 hours after topical application of either 0.5% epinastine cream than in the eyes of the placebo cream. Plus, Shoji et al. conducted an eight-week administration study on 0.5% epinastine cream in 124 patients aged 12 years or older with allergic conjunctivitis. They observed a significant decrease in the ocular itch score after cream administration [7]. The CAC test and the eight-week administration study suggested 0.5% epinastine cream as a useful medicine for carrying out proactive antihistamine medication, especially for patients having difficulty complying with eye drops multiple times. 

Some questions remain about the clinical application of 0.5% topical epinastine eyelid cream. First, the extent to which patients can comply with once-daily eyelid cream application in real-world practice is unknown. Plus, the trials selected asymptomatic patients with SAC. So, the effect of 0.5% topical epinastine eyelid cream on allergic conjunctivitis patients who are currently symptomatic and dependent on antihistamine eye drops has yet to be investigated. For example, it is unknown whether 0.5% topical epinastine eyelid cream can continuously suppress the fluctuation of itching for 24 hours after administration to symptomatic patients. It is also unclear whether it can maintain patients’ QOL well.

In this report, we prescribed 0.5% topical epinastine eyelid cream to symptomatic patients with PAC using antihistamine eye drops. Then, questionnaires were conducted on the changes in subjective perception of itching and QOL from immediately after medication administration up to 24 hours, as well as medication compliance. This study aims to assess medication compliance, 24-hour course of ocular itching, and daytime QOL of symptomatic allergic conjunctivitis patients who are dependent on antihistamine eyedrops, after switching to 0.5% topical epinastine eyelid cream. These surveys can potentially reveal the benefits of 0.5% topical epinastine eyelid cream in actual clinical practice for patients with active PAC.

## Materials and methods

This prospective study occurred at one clinical site: Tsukazaki Hospital, Hyogo, Japan. 

The study adhered to the ethical principles outlined in the Declaration of Helsinki. The Institutional Review Board in Tsukazaki Hospital reviewed and approved the study protocol. All patients provided written informed consent.

The inclusion criteria for this study were as follows: patients had to have been previously using antihistamine eye drops; have a diagnosis of ocular allergic disease based on physical findings; have keratoconjunctivitis scores lower than 2 according to the Japan Ocular Allergy Society Grading [1] by Miyazaki et al.; and be willing to switch their medication to 0.5% topical epinastine eyelid cream for allergy treatment.

Patients with uncontrolled keratoconjunctivitis or eyelid dermatitis were excluded. 

Patients applied their last dose of antihistamine eye drops on the morning of the first day they began using 0.5% topical epinastine eyelid cream. The cream was applied once-daily, and patients were advised to use it before bedtime. They discontinued all other eye drops except for artificial tear solutions.

Table 1 describes the questionnaire schedule. Details of the questionnaires are provided in Appendices A-C. Questionnaires were given before the start of 0.5% topical epinastine eyelid cream treatment and on the first and second days. Patients able to make frequent hospital visits completed additional questionnaires one week and one month after starting the eyelid cream.

**Table 1:** Schedule of the questionnaires. This table indicates the timing of each questionnaire administration. Before the eyelid cream application, we questioned patients' medication behavior and QOL under the usage of antihistamine eye drops. After the application of eyelid cream, we asked about their medication behavior, QOL, and ocular itch scores 24 hours after medication. QOL, quality of life

	Before the application	The first application	The second application	After weekly application	After the monthly application
Medication behavior	＊	＊	＊	＊	＊
Itching		＊	＊	＊	＊
QOLs	＊	＊	＊	＊	＊

During their outpatient visit, patients completed several questionnaires. These included one on compliance with medication (Appendix A), another on the time course of ocular itching from immediately before taking the drug until 24 hours after medication (Appendix B), and a third on daytime QOL (Appendix C).

We referred to the Japanese Allergic and Conjunctival Diseases Quality of Life Questionnaire (JACQLQ) version 1 [8] for the daytime QOL questionnaires. The total QOL score, calculated as the sum of the four individual QOL scores, was used for the statistical analysis described below.

To evaluate the efficacy of 0.5% topical epinastine eyelid cream in patients with perennial allergic conjunctivitis using antihistamine eye drops, ocular itch scores and QOL scores taken before, on the first day, and the second day of administration were compared by statistical analysis. We applied a mixed-effect model for repeated measures (MMRM) because this statistical method is less affected by repeated measurements and missing data. Total QOL scores taken before and on the first day and taken before and on the second day were compared. Similarly, ocular itch scores taken before and on the first day and taken before and on the second day were compared.

## Results

The 0.5% topical epinastine eyelid cream was introduced to 14 patients with perennial allergic conjunctivitis who were dependent on antihistamine eye drops. Eight of the 14 patients answered the questionnaire before starting the eyelid cream, as well as on the first and second days. The remaining six patients answered additional questions after one week and one month. A summary of the 14 cases is shown in Table 2. No adverse effects were observed in these patients. There were no notable differences in age, sex, or eye drop behaviors between patients followed for one month and those followed up to the second application.

**Table 2:** A summary of the 14 studied cases. F, female; M, male

Case	Age	Sex	Eyedrop medication	Eyelid cream medication	Follow-up
1	61	F	Regularly	Regularly	Second application
2	33	F	When feeling itchy	Regularly	Second application
3	53	F	Regularly	Regularly	Second application
4	53	F	Regularly	Regularly	Second application
5	18	M	Regularly	Regularly	Monthly application
6	58	F	Regularly	Regularly	Second application
7	74	M	Regularly	Regularly	Second application
8	71	M	Regularly	Regularly	Monthly application
9	24	F	Regularly	Regularly	Monthly application
10	44	F	Regularly	Regularly	Second application
11	24	F	When feeling itchy	Regularly	Monthly application
12	21	F	When feeling itchy	Regularly	Monthly application
13	70	F	Regularly	Regularly	Monthly application
14	24	F	Regularly	Regularly	Second application

Three patients - Case 2, Case 10, and Case 12 - reported using antihistamine eye drops as needed to relieve ocular itching on the questionnaires completed before eyelid cream administration. All 14 patients completed the proactive eyelid cream treatment. One patient mistakenly applied the eyelid cream twice on one occasion - once in the morning and again at bedtime. The remaining 13 patients applied 0.5% topical epinastine eyelid cream once before bedtime during the follow-up period. No patient skipped the eyelid cream application before bedtime.

**Figure 1:**
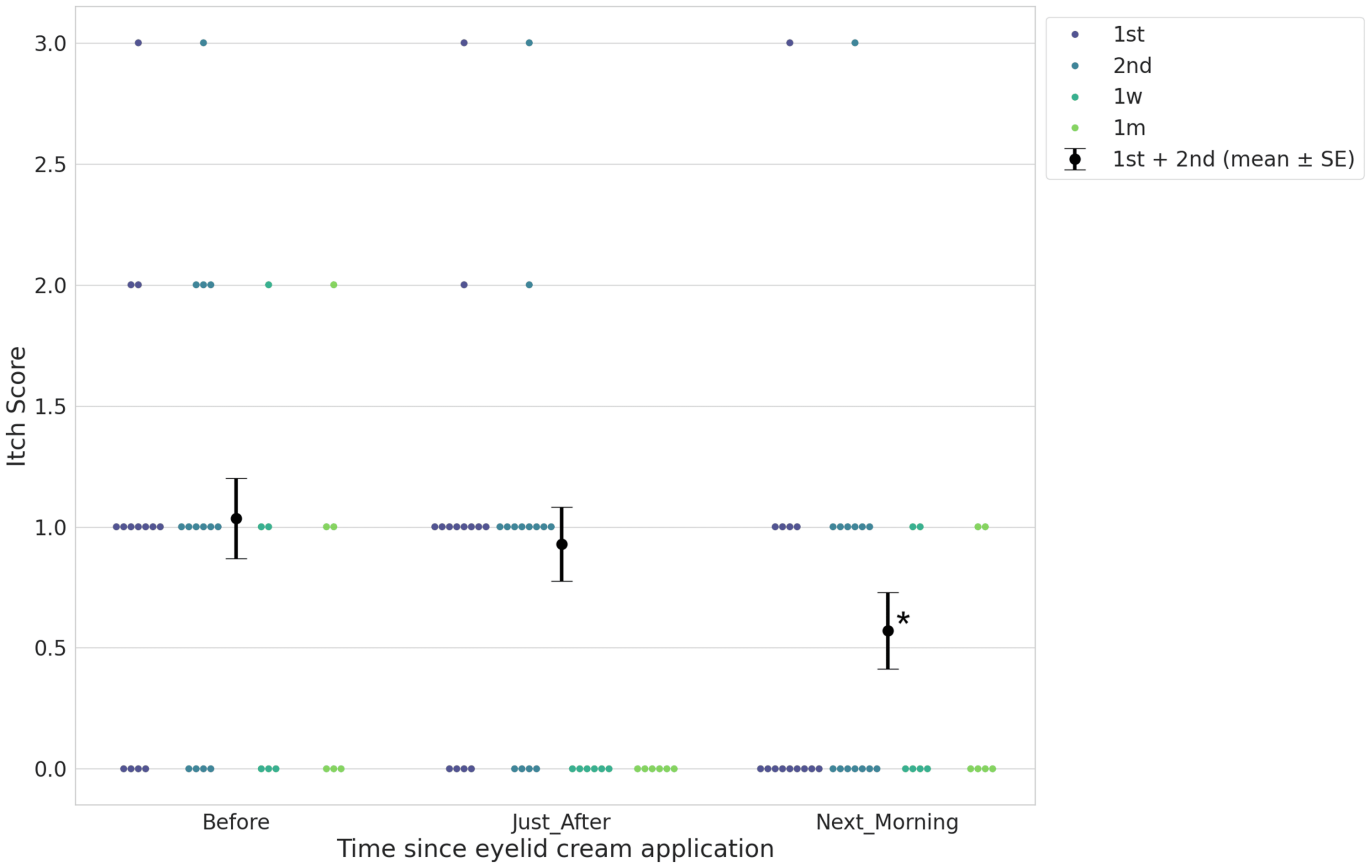
Twenty-four-hour transition in average ocular itch scores. A mixed-effect model for repeated measures was performed for statistical analysis and observed a significant difference in ocular itch scores before application and the next morning (＊, Figure 2, Table 4).

**Figure 2:**
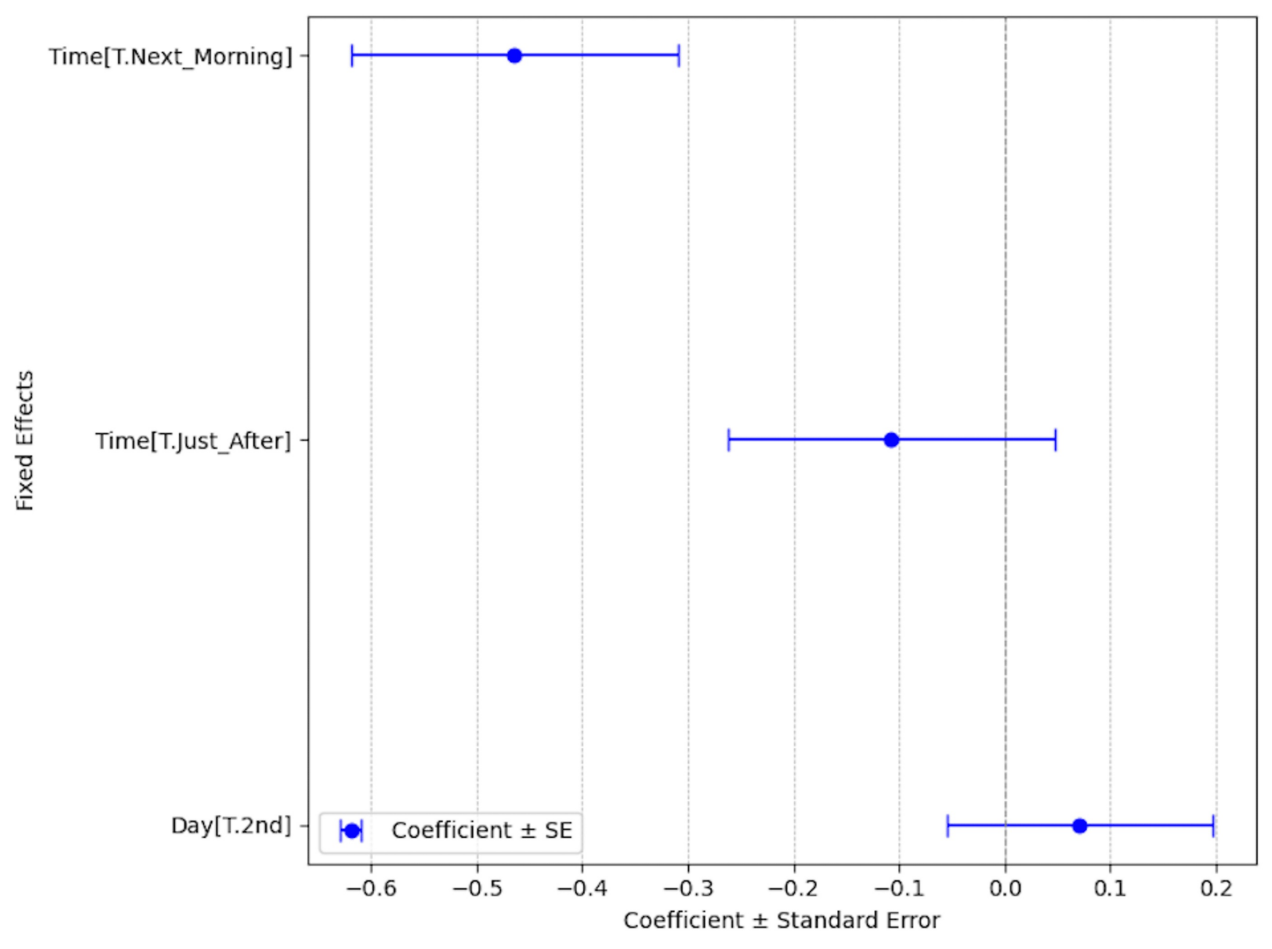
Mixed-effects model coefficients and standard errors for the 24-hour transition in average ocular itch scores. Time[T.Next_Morning]: The average ocular itch scores difference between before eyelid cream application and the next morning Time[T.Just_After]: The average ocular itch scores difference between before eyelid cream application and just after eyelid cream application Day[T.2nd]: The average ocular itch score difference between the first and second application

**Figure 3:**
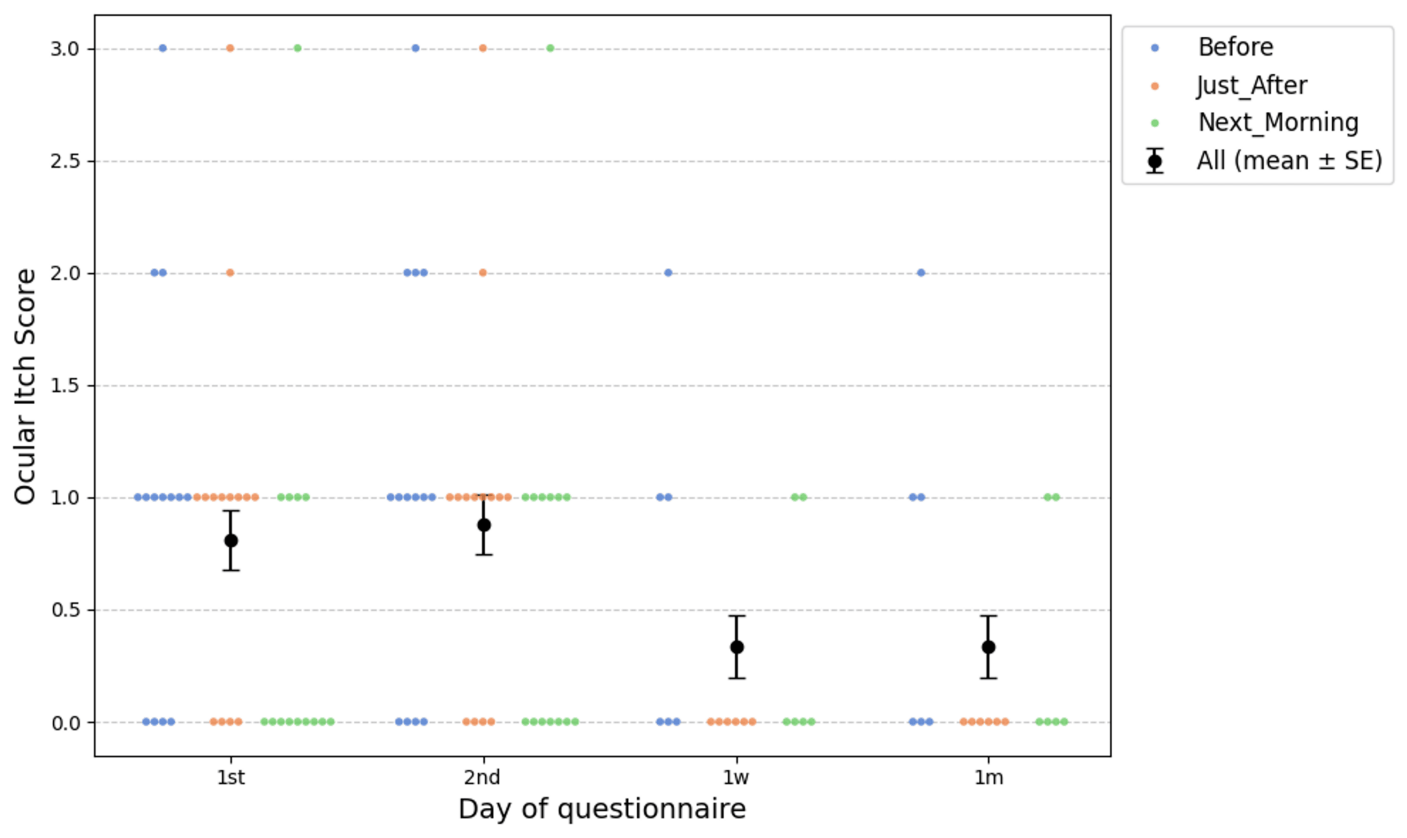
Monthly time course of average ocular itch scores, serialized by time after medication. All mean ± SE: mean scores ± standard error of ocular itch scores.

Table 3 shows the transition of ocular itching scores from immediately before starting the medication. Figure 1 presents ocular itch scores recorded on the same day in chronological order. The average ocular itch scores gradually decreased. Significant differences in mean ocular itch scores were detected between the time points before medication and the next morning (*P* = 0.003), but not between before medication and just after medication (*P* = 0.488), as determined by MMRM (Figure 2, Table 4). Figure 3 shows the transition of ocular itching scores segmented by the duration after medication. Although the scores increased at the time of the second application, overall, ocular itch scores gradually decreased.

**Table 3:** Summary of ocular itch scores. All 14 patients rated the severity of their eye itch just before and just after eyelid cream application and the next morning on a four-point scale: 0, no symptom, to 4, very severe. The questionnaire was held on the day of administration, the next day, after weekly application, and after the monthly application.

Case	First application			Second application			After the weekly application			After the monthly application		
	Before	Just after	Next morning	Before	Just after	Next morning	Before	Just after	Next morning	Before	Just after	Next morning
1	0	0	0	0	0	0						
2	2	1	1	2	1	1						
3	1	1	0	1	1	0						
4	1	1	0	2	1	1						
5	1	1	1	1	1	0	1	1	0	0	0	0
6	1	1	0	0	0	0						
7	0	0	0	0	0	0						
8	0	0	1	1	1	1	0	0	0	0	0	0
9	1	1	0	1	1	1	0	0	1	1	0	1
10	1	2	0	1	2	0						
11	2	1	3	2	1	3	2	1	2	2	0	1
12	1	1	1	1	1	1	1	1	1	1	0	0
13	0	0	0	0	0	0	0	0	0	0	0	0
14	3	3	0	3	3	1						

**Table 4:** Mixed-effects model coefficients with standard errors of ocular itch scores before medications to the second application. Coef., coefficient; Std.Err., standard error; CI [0.025, minimum end of 95% confidence interval CI 0.975], maximum end of 95% confidence interval; Day[T.2nd], the average ocular itch score difference between the first and second application; Time[T.Next_Morning], the average ocular itch scores difference between before eyelid cream application and the next morning; Time[T.Just_After], the average ocular itch scores difference between before eyelid cream application and just after eyelid cream application

	Coef.	Std.Err.	*z*-score	*P*-value	CI [0.025	CI 0.975]
Intercept	1	0.21	4.76	0	0.588	1.412
Day[T.2nd]	0.071	0.126	0.566	0.571	-0.176	0.319
Time[T.Just_After]	-0.107	0.155	-0.693	0.488	-0.41	0.196
Time[T.Next_Morning]	-0.464	0.155	-3.003	0.003	-0.767	-0.161
Group Variance	0.395	0.334				

**Figure 4:**
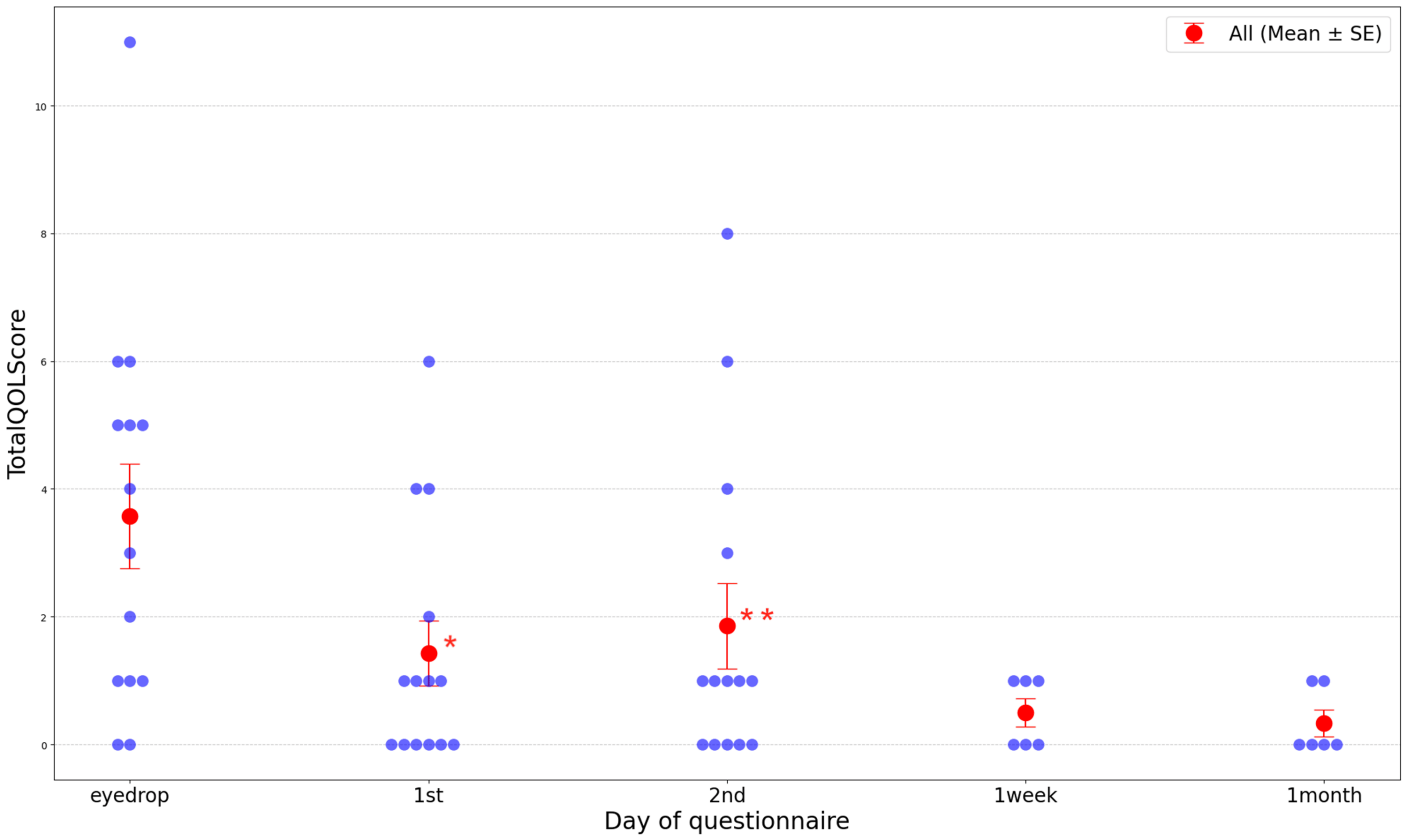
Monthly time course in total QOL scores. A mixed-effect model for repeated measures was performed for statistical analysis and observed a significant difference in the total QOL scores between before application and the first application (＊, Figure 5), and between before application and the second application (＊＊, Figure 5, Table 6). QOL, quality of life

**Figure 5:**
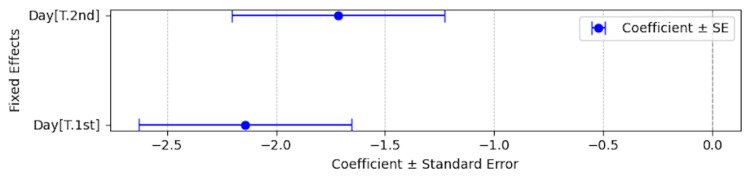
Mixed-effects model coefficients with standard errors for total QOL scores between before application and the first application, and between before application and the second application. Day[T.2nd]: Total QOL score difference between using eye drops and the second application Day[T.1st]: Total QOL score difference between using eye drops and the first application

Table 5 presents daytime QOL scores. Figure 4 describes the transition of the total QOL scores. Total QOL scores gradually decreased over time following the first administration. Significant differences in mean QOL scores were observed between before eyelid cream application and after the first application (*P* < 0.001), as well as between before application and after the second application (*P* < 0.001), according to MMRM analysis (Figure 5, Table 6), indicating improved QOL compared to when using antihistamine eye drops.

**Table 5:** A summary of QOL scores. Patients answered their complaints on a four-point scale: 0, no symptom, to 4, very severe. The questionnaire was held on the day of administration, the next day, after the weekly application, and after the monthly application. Interference: Interference with study/work/housework. QOL, quality of life

Case	Before the application				First application				Second application				After the weekly application				After the monthly application			
	Interference	Fatigue	Lethargy	Irritability	Interference	Fatigue	Lethargy	Irritability	Interference	Fatigue	Lethargy	Irritability	Interference	Fatigue	Lethargy	Irritability	Interference	Fatigue	Lethargy	Irritability
1	0	0	0	0	0	0	0	0	0	0	0	0								
2	2	0	0	3	2	1	0	1	2	0	0	1								
3	3	3	2	3	1	2	1	2	1	2	1	2								
4	0	1	1	3	0	0	1	1	1	1	1	1								
5	0	1	0	0	0	1	0	0	0	1	0	0	0	1	0	0	0	1	0	0
6	1	1	1	1	0	0	0	0	0	0	0	0								
7	0	1	0	1	0	0	0	0	0	0	0	0								
8	1	1	2	2	0	0	0	0	1	0	0	0	0	1	0	0	0	0	0	0
9	3	0	0	2	1	0	0	0	1	0	0	0	0	0	0	0	1	0	0	0
10	1	0	0	2	0	0	0	1	0	0	0	1								
11	1	0	0	0	1	0	0	0	1	0	0	0	1	0	0	0	0	0	0	0
12	1	0	0	0	0	0	0	0	0	0	0	0	0	0	0	0	0	0	0	0
13	0	0	0	0	0	0	0	0	0	0	0	0	0	0	0	0	0	0	0	0
14	2	1	1	2	1	1	1	1	2	2	2	2								

**Table 6:** Mixed-effects model coefficients with standard errors for total QOL scores from before medication to the second application. Coef., coefficient; Std.Err., standard error; CI [0.025, minimum end of 95% confidence interval; CI 0.975], maximum end of 95% confidence interval; Day[T.1st], total QOL score difference between using eye drops and the first application; Day[T.2nd], total QOL score difference between using eye drops and the second application QOL, quality of life

	Coef.	Std.Err.	*z*-score	*P-*value	CI [0.025	CI 0.975]
Intercept	3.571	0.677	5.272	<0.001	2.244	4.899
Day[T.1st]	-2.143	0.488	-4.392	<0.001	-3.099	-1.186
Day[T.2nd]	-1.714	0.488	-1.951	<0.001	-2.671	-0.758
Group variance	4.758	1.977				

## Discussion

This study examined medication compliance, 24-hour itch scores, and QOL when using 0.5% topical epinastine eyelid cream for symptomatic perennial allergic conjunctivitis after being prescribed epinastine eye drops.

In this study, all patients applied the eyelid cream before bedtime without fail during the follow-up period. We judged patients’ medication compliance as sufficient for the questionnaire of ocular itch scores and QOL scores to analyze. It is difficult to precisely assess medication compliance with the eyelid cream in this study, as over half of the patients completed questionnaires only two days after starting its use. Another long-term prospective cohort study with a large number of subjects would be necessary to compare medication compliance between eye drops and eyelid cream.

Ocular itch scores gradually decreased from before administration to the next morning without obvious fluctuation. The trend may reflect the process of epinastine diffusion from the eyelid cream into the eyelid tissue and conjunctiva. 

A past report supports the possible relation between the ocular itch transition in this study and the pharmacokinetics of epinastine eyelid cream. Mochizuki et al. applied 0.5% topical epinastine eyelid cream on the rabbit eyelid and measured epinastine concentration in the rabbit eyelid tissue a half an hour, 8 hours, and 24 hours after medication [5]. They observed that epinastine concentrations in the deeper parts of the eyelid sections and conjunctival layer increased at 8 and 24 hours post-dose compared to those at a half hour post-dose. Next, they studied pharmacokinetics in rabbit conjunctiva after epinastine cream or eye drops medication. The palpebral and bulbar conjunctivae of rabbits treated with 0.1% epinastine hydrochloride showed a rapid decrease in epinastine concentration, with no peak observed at 12 hours after administration. In contrast, those tissues in rabbits treated with 0.5% epinastine cream exhibited a peak in epinastine concentration at eight hours in the palpebral conjunctiva and at 12 hours in the bulbar conjunctiva, followed by a gradual decline. Although these experiments on animals cannot be applied to the pharmacokinetics in the human eyelid directly, similar behavior of epinastine molecules is expected in the human eyelid. It is assumed that epinastine concentration in the human eyelid was not enough for patients to feel ocular itch significantly alleviated just minutes after eyelid cream medication, and was enough 8-12 hours after eyelid cream medication, about the next morning, if patients applied the eyelid cream before bedtime. 

Although this questionnaire did not record detailed changes over time, ocular itch scores during 24 hours did not show obvious fluctuation. The possible reason is that the molecules of 0.5% topical epinastine eyelid cream diffused slowly through the eyelid to the conjunctiva and helped patients keep the epinastine concentration in the conjunctiva above a certain level.

Total QOL scores significantly decreased after applying 0.5% topical epinastine eyelid cream. In other words, QOL scores while using antihistamine eye drops were higher than those while using the eyelid cream. This result may be related to stabilizing epinastine concentration in the conjunctiva, followed by alleviating ocular itch fluctuation after switching from epinastine eye drops to epinastine eyelid cream.

Previous reports suggest the difficulty for SAC patients to control the fluctuation of ocular itches with antihistamine eye drops and the possible influence of this fluctuation on patients’ QOL deterioration. Fukagawa et al. showed the distribution of patients' frequency of ocular itching exacerbation, and about 83% of patients experienced unbearable exacerbations of ocular itching at least once-daily [2]. Plus, QOL scores gradually deteriorated as the frequency of itching increased.

Other previous reports also supported the possible effects of alleviating ocular itch fluctuation on patients’ QOL. Fukushima et al. compared patients with seasonal allergic conjunctivitis using antihistamine eye drops as needed and proactively, in aspects of allergic eye and nasal symptoms and QOL [3]. They reported that patients' allergic eye and nasal symptom scores did not display a significant difference, but patients’ QOL did. Although this study by Fukushima et al. did not monitor the frequency of allergic symptoms, this QOL improvement may be attributed to the stabilization of drug concentration in the conjunctival sac, thanks to proactive eye drop medication.

Our study did not take questionnaires about the ocular itch time course when using eye drops, so we cannot make a precise comparison in daytime ocular itch fluctuation between when using eye drops and when using eyelid cream. However, considering the implications of past reports, improving QOL scores after switching from epinastine eye drops to epinastine eyelid cream may be caused by the control of ocular itch fluctuation after eyelid cream administration.

The study had the following limitations. We took questionnaires about the QOL scores and medication behavior before eyelid cream administration, when applying eye drops - only once, just before administration, and we did not take questionnaires about the 24-hour ocular itch fluctuation before eyelid cream administration. Therefore, it is difficult to make a precise assessment of the change in the QOL scores, quality of ocular itch, and medication behavior due to switching medication from eye drops to eyelid cream. It is desirable to conduct another survey of sufficient duration, both in eye drop use and in cream use, to allow for more accurate comparisons. Ideally, trials that allow comparison between the two groups - cross-over trials and cohort trials, for example - are desirable.

## Conclusions

We prescribed 0.5% topical epinastine eyelid cream to symptomatic patients with PAC using antihistamine eye drops. Ocular itches gradually decreased 24 hours after eyelid cream application, and their QOL scores significantly improved after eyelid cream application. The 0.5% topical epinastine eyelid cream has the potential to minimize the fluctuation of ocular allergic symptoms and improve the QOL of patients with PAC.

## Appendices

Appendix A

**Table 7:** Questionnaires of patients’ medication behavior: Please tell us about the medication you used from yesterday to today. Please circle the applicable items regarding frequency and timing.

Frequency	0 times	1 time	2 times	3 or more times
Timing	When itching bothers me		At roughly fixed times	

Appendix B

**Table 8:** Questionnaires of 24-hour transition of ocular itches: How did your eye itching change during the 24 hours from applying the cream to your eyelids until applying the new cream the next day? Please circle the number that best represents the severity of eye itching at each time point.

	0: No symptoms	1: Mild	2: Moderate	3: Severe	4: Very Severe
Before medication	0	1	2	3	4
After 2-3 minutes	0	1	2	3	4
Next morning upon waking	0	1	2	3	4

Appendix C

**Table 9:** Questionnaires of daytime quality of life: Did eye itching interfere with your daily life? Please circle the number that best describes how eye itching impacted your daily life.

	0: No symptoms	1: Mild	2: Moderate	3: Severe	4: Very Severe
Interference with study/work/housework	0	1	2	3	4
Fatigue	0	1	2	3	4
Lethargy	0	1	2	3	4
Irritability	0	1	2	3	4
